# Efficacy and Safety in a Real-World Study of the New Oral Formulation of Semaglutide in Patients with Chronic Kidney Disease and Type 2 Diabetes Mellitus

**DOI:** 10.3390/jcm13175166

**Published:** 2024-08-30

**Authors:** María Marques Vidas, Paula López-Sánchez, Paula Sánchez-Briales, María Victoria López Illazquez, Jose Portolés

**Affiliations:** 1 Nephrology Department, Hospital Universitario Puerta de Hierro Majadahonda, IDIPHISA, 28222 Madrid, Spain; nefro_metodologia@yahoo.com (P.L.-S.); paulasanchezbriales@gmail.com (P.S.-B.); mlopezillazquez@salud.madrid.org (M.V.L.I.); josem.portoles@salud.madrid.org (J.P.); 2 Medicine Department, Facultad de Medicina, Universidad Autónoma de Madrid, IDIPHISA, 28029 Madrid, Spain

**Keywords:** type 2 diabetes mellitus, chronic kidney disease, semaglutide, glucagon peptide type 2 receptor agonists, glycosylated hemoglobin

## Abstract

**Background/Objectives:** GLP-1 receptor agonists (GLP-1RAs) have emerged as fundamental components in the treatment of type 2 diabetic patients (T2DM) with chronic kidney disease (CKD). The oral formulation represents a novel therapeutic tool but may affect drug efficacy. This study sought to compare the effectiveness of subcutaneous versus oral semaglutide formulations in patients with CKD. **Methods:** A retrospective study in a real-world setting compared type 2 diabetes and chronic kidney disease patients, initiating oral semaglutide treatment to a historically matched control group treated with subcutaneous semaglutide. The matching considered factors such as estimated glomerular filtration rate (eGFR), age, and sex. **Results:** Nineteen patients were included in both groups, with a mean age of 68.0. Seventy-two percent were males with a CKD-EPI eGFR of 49.9 mL/min/1.73 m^2^ and a median urine albumin-to-creatinine ratio of 12.7 mg/g. Of the study participants, 94% and 79% of patients were on the maximum semaglutide sbc vs. oral dose, while 5.3% and 15.8% were on the sbc vs. oral low dose. Oral semaglutide significantly reduced HbA1C and BMI, identical to the control group (−0.9 and −1.4, *p* > 0.05). Renal function parameters and blood pressure remained stable throughout the follow-up in both groups. The main side effect was digestive intolerance (affecting three patients in the oral group and two patients in the subcutaneous group, *p* = 0.6), although the treatment abandonment percentage was similar. **Conclusions:** The oral formulation of semaglutide demonstrated equivalent effectiveness in glucose control and body weight management in patients with T2DM and CKD, even with a higher proportion of patients receiving low to medium doses. Gastrointestinal side effects were comparable between both oral and subcutaneous formulations.

## 1. Introduction

Type 2 diabetes mellitus (T2DM) stands out as a significant contributor to end-stage renal disease requiring renal replacement therapy [1]. Relevant clinical trials have shown that glucagon-like peptide-1 receptor agonists (GLP-1 RAs) achieve substantial reductions in predefined renal outcomes. The LEADER clinical trial notably highlighted the efficacy of liraglutide in mitigating renal complications, including new-onset proteinuria, doubling serum creatinine, end-stage renal disease (ESRD), and renal death [2]. Building on previous studies, the SUSTAIN-6 trial [3] evaluated the impact of semaglutide treatment in patients diagnosed with ESRD stages 3–5. The SUSTAIN-6 trial unveiled a similar reduction in combined renal outcomes, encompassing new or worsening nephropathy characterized by persistent albuminuria, doubling serum creatinine and creatinine clearance below 45 mL/min/1.73 m^2^. The results from clinical trials of dulaglutide, including AWARD and REWIND, were promising, showcasing beneficial effects on albuminuria and the preservation of the glomerular filtration rate (GFR) [4,5]. Lastly, the FLOW clinical trial [6] demonstrated a significant reduction in the risk of kidney disease progression in patients treated with subcutaneous semaglutide compared to the placebo group. These results included a lower incidence of sustained eGFR decline, end-stage kidney disease, and renal death.

It is important to note that semaglutide represents the first GLP-1 RA to introduce an oral formulation. The PIONEER 5 clinical trial [7] further underscores the effectiveness of oral semaglutide in reducing body weight and glycosylated hemoglobin (HbA1C) in type 2 diabetes mellitus and chronic kidney disease (T2DM-CKD) patients with a GFR range of 30–60 mL/min/1.73 m^2^.

However, renal impairment can affect various pathways of drug metabolism within the gut and is associated with other changes, including alterations in drug absorption, plasma protein binding, drug transport, and tissue distribution [8]. While prior studies have suggested that renal impairment has no significant impact on oral semaglutide pharmacokinetics [9], there has been a notable absence of a comparative analysis of efficacy and safety between oral and subcutaneous semaglutide formulations within the CKD population.

We present this retrospective, proof-of-concept study to evaluate the efficacy and safety of the oral formulation compared to the subcutaneous formulation of semaglutide in a population of T2DM-CKD patients.

## 2. Materials and Methods

This retrospective, single-center, real-world study was conducted on patients with T2DM-CKD, initiated on oral semaglutide. The aim was to analyze the efficacy of glycemic control and the effect on weight in both the oral and subcutaneous formulations (Rybelsus, Novo Nordisk, Coppenhage, Denmark; Ozempic, Novo Nordisk). The impact on glomerular filtration and albuminuria, as well as the occurrence of adverse effects and the rate of treatment discontinuation, was examined.

### 2.1. Inclusion and Exclusion Criteria

Patients were selected from our unit’s database using the following inclusion and exclusion criteria:

- Inclusion criteria: we included patients, aged >18 years with T2DM and with an estimated glomerular filtration rate (eGFR) using the Chronic Kidney Disease Epidemiology Collaboration (CKD-EPI) formula >15 mL/min/1.73 m^2^, who were being followed in our nephrology outpatient clinic.
- Exclusion criteria: Patients with kidney transplants, patients with CKD of non-diabetic etiology, patients with CKD stage 5, and patients participating in clinical trials were excluded from the study. Patients who had previously received other GLP-1 RA treatments were also excluded.

**Figure 1:**
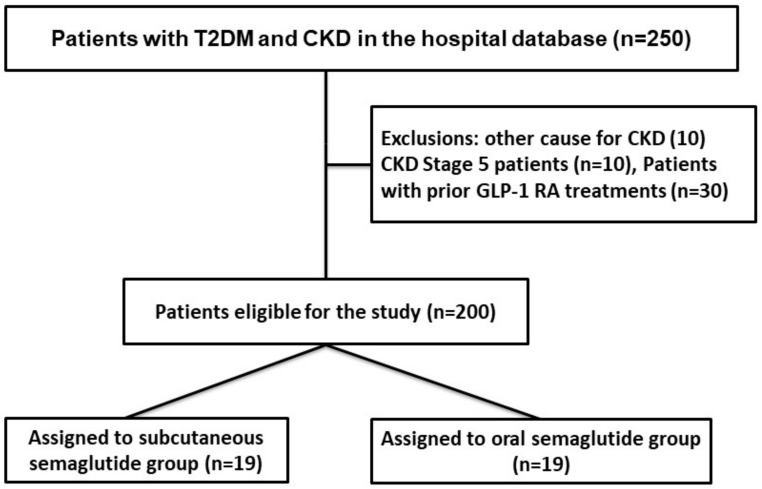
Flowchart of cohort selection for study comparing subcutaneous and oral semaglutide in patients with type 2 diabetes mellitus and chronic kidney disease.

The inclusion period was January 2017–December 2022. A historical cohort, comprising patients treated with subcutaneous semaglutide, served as the control group (see the flowchart in Figure 1). These patients were selected from our database and carefully matched with the study group based on eGFR, age, and sex.

Semaglutide dosage was up-titrated according to standard recommendations, typically every month unless there was digestive intolerance. However, due to accessibility issues of outpatient nephrology clinics during the COVID-19 pandemic, the up-titration was delayed in some patients receiving the the oral formulation.

The dosages for both oral and subcutaneous semaglutide were as follows:

Oral semaglutide doses:

- Low dose: 3 mg/day (used by 15.8% of patients)
- Medium dose: 7 mg/day (used by 5.3% of patients)
- High dose: 14 mg/day (used by 78.9% of patients)

Subcutaneous semaglutide doses:

- Low dose: 0.25 mg/week (used by 5.3% of patients)
- High dose: 1.0 mg/week (used by 94.7% of patients)

These doses were administered over the course of the study, and the proportion of patients receiving each dose was reported at the 6th month or at the end of follow-up. None of the patients were on the medium sbc dose (0.5 mg/week) by the end of the study.

### 2.2. Collected Data

Data were collected at baseline, 3 months, and 6 months, including body weight, body mass index (BMI), eGFR assessed using the CKD-EPI equation [10], urinary albumin-to-creatinine ratio (UACR), HbA1C, and lipid profile. Information on antihypertensive and antihyperglycemic treatments was also recorded. Cardiovascular risk factors were documented during the baseline visit, and secondary events were monitored. The study protocol was approved by the Ethical Committee of the Hospital Universitario Puerta de Hierro [ref 61/23].

### 2.3. Statistical Analysis

Descriptive analysis was conducted, including measurements of central tendencies, dispersion, and position for quantitative variables, as well as the frequency distribution for qualitative variables. Paired sample tests were employed for quantitative variables, while McNemar’s test was used to study the differences between paired qualitative variables. A generalized linear model was applied to identify differences in quantitative variables between baseline values and values at 12 months. Statistical significance was set at *p* < 0.05. All statistical analyses were performed using STATA 16.1 (Stata Statistical Software, StataCorp LLC, College Station, TX, USA).

The STROBE checklist for cohort studies [11] was utilized to ensure the comprehensive reporting of the study design, implementation, and results. (The checklist is included in the Supplementary Materials.)

## 3. Results

The characteristics of the treatment group with oral semaglutide and the control group with subcutaneous semaglutide are shown in Table 1. Most of the study cohort exhibited stage 3 CKD, with a specific prevalence of 34.2% in stage 3b (CKD-EPI stage 2: 23.7% (n = 9), stage 3: 63.2% (n = 24), stage 4: 13.2% (n = 5)). The cause of CKD was primarily attributed to T2DM. As shown in the table, there were no significant differences in the demographics or comorbidity parameters between the study population and the selected control group.

The mean dosage and the percentage of patients upon each presentation of both formulations are shown in Table 2. A significant number of patients remained on the lower dosages of the oral formulation, mainly due to the spacing out of appointments during the COVID-19 pandemic.

**Figure 2:**
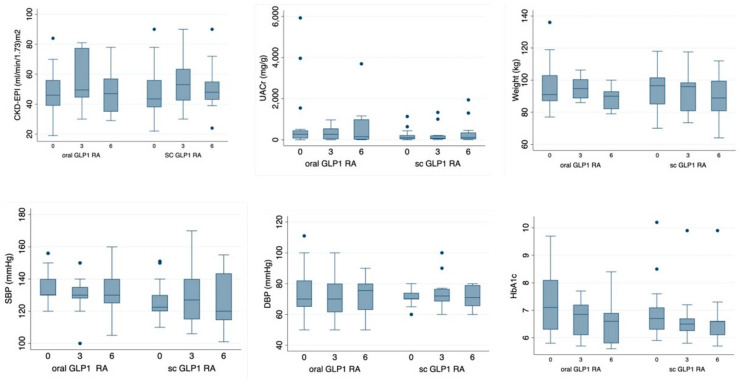
Box plot of the main clinical variables in the study groups.

The clinical outcomes of the two study groups are presented in Table 3 and Table 4, and in Figure 2.

We observed a decreased UACR in both groups (Table 3 and Table 4), although the decrease did not reach statistical significance. The eGFR experienced a slight increase at three months in both groups, which was only sustained at six months in the subcutaneous semaglutide group. The dispersion of the data related to eGFR and UACR, as shown in Figure 2, may have influenced the absence of significant changes throughout the study.

Both groups had good baseline glycemic control and, despite the semaglutide formulation, HbA1C levels showed a similar decrease. However, the decline was slightly more significant in the oral formulation (−0.9 [from −1.5 to −0.4] vs. −0.4 [from −0.8 to −0.3], *p* < 0.2) (Table 4). We observed a significant decrease in weight and body mass index (BMI) in both groups at six months (Table 4), with no significant differences between the two formulations.

There were no significant differences in blood pressure values throughout the follow-up. Three (15.8%) and two (10.5%) patients experienced digestive adverse effects in the oral semaglutide and subcutaneous semaglutide groups, respectively (*p*-value = 0.6). There were three treatment discontinuations in the oral semaglutide group and one in the subcutaneous semaglutide group (Table 4). In the oral semaglutide group, all discontinuations were due to digestive intolerance, while in the control group, the discontinuation was due to the onset of another pathology (COVID-19).

## 4. Discussion

In this study examined here, we have demonstrated that using the oral formulation of semaglutide in patients with different stages of CKD achieves very similar results in terms of glycemic control and weight loss compared to the subcutaneous formulation. These results persist despite the difficulty that could initially be inferred from adherence to stringent dosing guidelines to optimize intestinal absorption. The oral formulation of semaglutide also showed a safety profile similar to that of the subcutaneous formulation, with gastrointestinal intolerance as the main adverse effect in a similar range, regardless of the drug formulation.

Glucagon-like peptide-1 receptor agonists (GLP-1 RAs) collectively exhibit efficacy in reducing both glucose levels and body weight, demonstrating a diminished risk of hypoglycemia; current KDIGO (Kidney Disease Improving Global Outcomes) and ADA (American Diabetes Association) guidelines prioritize this pharmacological class of antihyperglycemic drugs in the leading positions for the treatment of patients with T2DM and CKD [12]. Beyond their efficacy as antihyperglycemic agents, GLP-1 RAs have demonstrable cardiovascular and renal benefits, strengthening the recommendation of using this therapeutic class in individuals with T2DM at high cardiovascular risk [13].

As mentioned in the introduction, there is already a significant body of literature regarding the efficacy and safety of GLP-1 RAs in the CKD population. The development of an orally administered GLP-1 receptor agonist (GLP-1 RA) faced significant challenges due to the molecule’s susceptibility to gastrointestinal degradation and poor absorption across the intestinal wall. However, these issues were overcome with the introduction of sodium N-[8-(2-hydroxybenzoyl) amino] caprylate (SNAC) technology. SNAC enhances the absorption of semaglutide through the stomach lining, effectively allowing for oral administration of this GLP-1 RA [14]. Despite this technology, to achieve adequate absorption of the oral formulation of semaglutide, it must be administered on an empty stomach, with a small amount of water, and without the ingestion of any liquid, food, or medication for the next 30 min [15].

Although the efficacy and safety of oral semaglutide were evaluated in a population with stages 3–5 chronic CKD in the PIONEER 5 study [16], a comparison of the efficacy and safety of both formulations in this population was yet to be evaluated.

The pharmacokinetics of oral drugs are affected in CKD, with delayed gastric emptying, increased gastric pH, and gastrointestinal edema known to decrease drug oral absorption and bioavailability [17].

To address this issue, Granhall et al. [9] conducted an interesting clinical trial testing the pharmacokinetics of oral semaglutide in populations with different degrees of CKD, finding that there were no significant differences in the maximum concentrations of the drug or sodium N-[8-(2-hydroxybenzoyl) amino] caprylate (SNAC) technology, as well as in the area under the curve (AUC) with regards to renal function. However, in this clinical trial, the drug was only administered for 10 days, the administration was monitored, and no medication was given until 2 h had passed; these circumstances differ significantly from the routine recommendations regarding dosage (30 min intervals before any food or medication) without considering the possible deviations from the optimal administration pattern in real life.

The findings of our study align with those observed in the SUSTAIN-6 [3] and PIONEER 5 [7] trials, which demonstrated that semaglutide, whether administered subcutaneously or orally, is effective in reducing HbA1c and body weight in patients with type 2 diabetes mellitus (T2DM) and chronic kidney disease (CKD). Specifically, our study corroborates the efficacy of semaglutide in maintaining stable renal function, as reflected in the stable eGFR and UACR levels observed, similar to the outcomes reported in the FLOW study [6]. Furthermore, our results show comparable gastrointestinal side effects across both formulations, which is consistent with the safety profiles documented in the broader literature. However, it is noteworthy that the proportion of patients requiring lower doses due to gastrointestinal intolerance was higher in the oral group, which is potentially attributable to delayed up-titration during the COVID-19 pandemic.

This work has several limitations: (1) it is a single-center study, so the reproducibility of the results in another setting cannot be guaranteed; (2) the small sample size in our study may limit the generalizability of the findings; (3) it is a retrospective but non-randomized study that used a historical control group—although there were no significant changes in the standard of care for this type of patient during the study period, we cannot rule out the existence of a temporal bias; and (4) patients who had previously been on another GLP-1 RA and started treatment with oral semaglutide were excluded from this analysis, so we cannot rule out that this type of population may behave differently. Finally, (5) the evaluation of renal function was carried out with single samples of sCr and albumin-to-creatinine ratio, which did not allow us to calculate the eGFR slope. This seems to be a more appropriate method to evaluate the progression of renal function. This evaluation also did not allow us to overcome the variability of albuminuria, though the progression of eGFR was not the primary objective of this study.

However, several strengths of this work deserve to be highlighted. First, this study addresses a pertinent clinical question about the efficacy and safety of oral semaglutide, a new therapeutic option, in a population with T2DM and CKD; this contributes to the emerging field of diabetes management in patients with renal impairment. Second, the research design—a real-world study—adds to this study’s validity, and the inclusion of a historical control group matched for eGFR, age, and sex strengthens the comparative analysis. Third, this study not only examines the primary outcomes of HbA1c and BMI reduction, as performed in previous randomized clinical trials, but also evaluates renal function parameters, blood pressure, and side effects, therefore providing a comprehensive assessment of the treatment’s effectiveness and safety. Finally, this study’s focus on real-world applicability, considering typical patient populations and treatment conditions, enhances the generalizability of these findings.

## 5. Conclusions

This exploratory real-world study suggests that the oral formulation of semaglutide represents an alternative at least as effective as the subcutaneous formulation in patients with type 2 diabetes and renal disease. The oral formulation also demonstrates a good safety profile that is very similar to the subcutaneous formulation.

## Figures and Tables

**Table 1:** Demographic data at baseline.

	Total	Oral	Subcutaneous	*p*-Value
N	38	19	19	
Age at T2DM diagnose (y)	55.8 (8.4)	56.4 (7.3)	54.9 (9.1)	0.6
Age at initiating GLP-1 RA (y)	68.4 (9.5)	68.6 (7.4)	67.4 (11.1)	0.7
Men (%)	73.7	73.7	73.7	1.0
Cause of renal disease (%)				1.0
CKD + T2DM	89.5	89.5	89.5	
Other	10.5	10.5	10.5	
Cardiovascular risk factors (%)				
Hypertension	94.8	100	89.5	0.2
Dyslipemia	65.8	79	52.6	0.09
Smoker	50.0	52.6	47.4	0.8
Ischemic cardiopathy	2.7	0	5.3	0.3
Ictus	5.5	5.6	5.3	1
Peripheral arterial disease	5.3	5.3	5.3	1
Follow-up (month)	6 [3–6]	6 [3–6]	6 [6–6]	0.1
Lost to follow-up or treatment discontinuation (%)	13.2 (5/38)	21.1 (4/19)	5.3 (1/19)	0.2
Renal function parameters				
sCr (mg/mL)	1.5 (0.4)	1.5 (0.4)	1.5 (0.4)	0.9
eGFR (mL/min/1.73 m^2^)	48.5 (19.1)	48.0 (18.8)	48.9 (19.9)	0.9
eGFR 60–90 (%)	23.7	21.1	26.3	0.9
eGFR 45–60 (%)	34.2	31.6	36.8	
eGFR 30–45 (%)	29.0	31.6	26.3	
eGFR < 30 (%)	13.2	15.8	10.5	0.9
UACR (mg/g)	119.1 [34.6–334.2]	268.5 [66.2–449.1]	101.8 [27.8–219.4]	0.003
HbA1C (%)	7.0 (0.9)	7.2 (1.1)	6.7 (0.7)	0.2
Other clinical data				
BMI (kg/m^2^)	33.2 (3.7)	33.4 (3.5)	33.0 (4.0)	0.8
Weight (kg)	94.9 (13.5)	96.7 (14.5)	93.2 (12.7)	0.4
Systolic blood pressure (mmHg)	130.7 (11.5)	134.8 (10.0)	126.7 (11.9)	0.04
Diastolic blood pressure (mmHg)	72.8 (12.1)	74.7 (16.0)	70.9 (6.2)	0.3

Footnote: T2DM: type 2 diabetes mellitus; y: years; BMI: body mass index, CKD: chronic kidney disease, sCr: serum creatinine; eGFR: estimated glomerular filtration rate, GLP-1 RA: glucagon-like peptide type 1 receptor agonist, HbA1C: glycated hemoglobin, UACR: urinary albumin to creatinine ratio. We performed the *t*-student test, Wilcoxon rank-sum test, or chi-square assessment.

**Table 2:** GLP1-RA dosage at the 6th month or end of follow-up.

		Oral (mg/Day)	Subcutaneous (mg/Week)	*p*-Value
Dosage at 6th Month/End of Follow-Up		11.9 (4.3)	1.0 (0.2)	NA
Dosage at 6th Month (percentage)	Low	3 mg/15.8%	0.25 mg/5.3%	0.3
	Medium	7 mg/5.3%		
	High	14 mg/78.9%	1.0 mg/94.7%	

Footnote: GLP-1 RA glucagon-like peptide type 1 receptor agonist; mg: milligram; NA: not applicable.

**Table 3:** Changes in clinical and analytical parameters at baseline, 3 months, and 6 months.

Time (Month)	0	3	6	0	3	6
	Oral			Subcutaneous		
N	19	13	11	19	12	14
sC (mg/mL)	1.5 (0.4)	1.4 (0.4)	1.6 (0.4)	1.5 (0.4)	1.4 (0.3) **	1.3 (0.5)
eGFR (mL/min/1.73 m^2^)	48.0 (18.8)	56.4 (18.0)	48.8 (16.3)	48.9 (19.9)	53.9 (16.5) **	53.4 (18.6) *
UACR (mg/g)	268.5 [66.2–449.1]	261.0 [30.0–544.9]	230.8 [14.0–1156.8]	101.8 [27.8–219.4]	70.1 [32.7–199.1]	88.4 [20.2–128.0]
BMI (kg/m^2^)	33.4 (3.5)	31.7 (1.9)	30.2 (1.6)	33.0 (4.0)	32.3 (4.5)	32.2 (3.3)
Weight (kg)	96.7 (14.5)	95.2 (7.3) *	90.4 (8.0) ***	93.2 (12.7)	92.4 (13.8) *	88.4 (7.6) *
SBP (mmHg)	134.8 (10.0)	130.1 (12.0)	135.6 (13.6)	126.7 (11.9)	124.3 (15.8)	131.2 (20.4)
DBP (mmHg)	74.7 (16.0)	72.0 (13.3)	74.4 (12.4)	70.9 (6.2)	72.2 (7.4)	70.4 (7.1)
HbA1C (%)	7.2 (1.1)	6.7 (0.7) *	6.4 (1.0) ***	6.7 (0.7)	6.7 (1.1)	6.5 (0.5)

Footnote: BMI: body mass index, DBP: diastolic blood pressure, SBP: systolic blood pressure, eGFR: estimated glomerular filtration rate, HbA1C: glycated hemoglobin, sC: serum creatinine, UACR: urinary albumin to creatinine ratio. Paired *t*-test * *p* < 0.05; ** *p* < 0.01; *** *p* = 0.001.

**Table 4:** Clinical efficacy and adverse effects of both semaglutide formulations.

Difference (Final-Basal)	Oral	Subcutaneous	*p*-Value
N	11	14	
sCr (mg/mL)	0.02 [−0.04 to 0.1]	−0.07 [−0.2 to 0]	0.1
eGFR (mL/min/1.73 m^2^)	−2 [−6 to 0]	4 [0–8]	0.001
UACR (mg/g)	−16.3 [−272.3 to 4.4]	5.9 [2.7 to 8.8]	0.1
UACR reduction (%)	−14.0 [−78.9 to 157.6]	−13.2 [−41.7 to −27.3]	
BMI (kg/m^2^)	−1.4 [−3.6 to −1.2]	−1.0 [−1.3 to 0.6]	0.2
Weight (kg)	−4.5 [−10 to −4]	−4 [−8 to −2]	0.4
SBP (mmHg)	−7 [−15 to 0]	0 [0 to 0]	0.3
DBP (mmHg)	0 [−16 to 0]	0 [−2 to 0]	0.9
HbA1C % (SD)	−0.9 [−1.5 to −0.4]	−0.4 [−0.8 to −0.3]	0.2
Adverse events	3	1	0.3
Gastrointestinal (%)	3 (16%)	0	0.07
Other causes (%)	0	1 (5%)	0.6

Footnote: DBP: diastolic blood pressure, sCr: serum creatinine, eGFR: estimated glomerular filtration rate, UACR: urinary albumin to creatinine ratio, BMI: body mass index, HbA1C: glycated hemoglobin, SBP: systolic blood pressure.

## Data Availability

Data will be available after a formal request to the corresponding author.

## Supplementary Materials

The following supporting information can be downloaded at: https://www.mdpi.com/article/10.3390/jcm13175166/s1, File S1: STROBE checklist.
