# Supplementary material for: Efficacy and Safety in a Real-World Study of the New Oral Formulation of Semaglutide in Patients with Chronic Kidney Disease and Type 2 Diabetes Mellitus
Source: J Clin Med. 2024 Aug 30;13(17):5166. doi: 10.3390/jcm13175166 (PMC11396478; doi:10.3390/jcm13175166)
Supplement: Supplementary file 1 [file jcm-13-05166-s001.zip › jcm-3113305-supplementary.pdf]

File S1: STROBE checklist

1. Title and Abstract

- **1a. Title:** Does the title clearly indicate that this is a cohort study?
  - **Answer:** Yes, the title clearly indicates that this is a cohort study by mentioning it as a "real-world" study.
- **1b. Abstract:** Does the abstract provide key information on study design, participants, exposure, comparator, outcomes, and conclusions?
  - **Answer:** Yes, the abstract covers the key aspects mentioned, including the retrospective design, participants, exposures (semaglutide formulations), outcomes (efficacy in glycemic control and weight), and conclusions.

2. Introduction

- **2a. Background/Rationale:** Does the introduction explain the scientific background and provide justification for the study?
  - **Answer:** Yes, the introduction provides adequate background on the importance of GLP-1RAs in patients with T2DM and CKD and justifies the need to compare oral and subcutaneous semaglutide formulations.
- **2b. Objectives:** Are the study objectives clearly defined?
  - **Answer:** Yes, the objectives are clearly defined as the comparison of efficacy and safety of the two semaglutide formulations.

3. Methods

- **3a. Study Design:** Is the study design (prospective or retrospective cohort) clearly described?
  - **Answer:** Yes, it is described as a retrospective real-world study.
- **3b. Setting:** Are inclusion and exclusion criteria, and the process of selecting participants, clearly described?
  - **Answer:** Yes, inclusion and exclusion criteria are well-defined, and the selection process for each group is explained.
- **3c. Variables:** Are exposure and outcome variables clearly defined?
  - **Answer:** Yes, the main variables (HbA1c, BMI, eGFR, UACR, etc.) are clearly defined.
- **3d. Data Sources/Measurement:** How were data obtained and measurements conducted?
  - **Answer:** Data were obtained from electronic medical records, and standard laboratory measurements were performed.
- **3e. Bias:** Are potential sources of bias identified and addressed?
  - **Answer:** Limitations such as sample size, temporal bias and lack of randomization are mentioned.
- **3f. Study Size:** How was the sample size determined?
  - **Answer:** The method for determining the sample size is not detailed, though it is noted that 19 patients were included in each group.
- **3g. Quantitative Variables:** Are statistical methods used to analyze the data clearly described?
  - **Answer:** Yes, the statistical methods used, including paired sample tests and generalized linear models, are clearly described.

4. Results

- **4a. Participants:** Is the number of participants at each stage of the study clearly described?
  - **Answer:** Yes, the number of participants at the start and during follow-up, including dropouts, is clearly described.
- **4b. Descriptive Data:** Are baseline characteristics of participants provided?
  - **Answer:** Yes, demographic and clinical characteristics of the participants are detailed in a table.
- **4c. Outcome Data:** Are the main study results presented?
  - **Answer:** Yes, results on HbA1c, BMI, eGFR, UACR, and other parameters are presented.

5. Discussion

- **5a. Key Results:** Are the key results interpreted in the context of the study's strengths and limitations?
  - **Answer:** Yes, several limitations and strengths of the study are discussed.
- **5b. Limitations:** Are the limitations of the study clearly addressed?
  - **Answer:** Yes, limitations such as sample size, being a single-center study and using a historical control group are discussed.
- **5c. Interpretation:** Are the results interpreted in the context of the current evidence?
  - **Answer:** Yes, the results are interpreted by comparing them with previous studies.
- **5d. Generalizability:** Is the generalizability (external validity) of the study results discussed?
  - **Answer:** Yes, the applicability of the findings in clinical practice is mentioned.

6. Other Information

- **6a. Funding:** Are sources of funding and any conflicts of interest disclosed?
  - **Answer:** Yes, funding sources are disclosed, and no conflicts of interest are reported.
